# Structural Insights into the Effector – Immunity System Tse1/Tsi1 from *Pseudomonas aeruginosa*

**DOI:** 10.1371/journal.pone.0040453

**Published:** 2012-07-06

**Authors:** Juliane Benz, Christina Sendlmeier, Thomas R. M. Barends, Anton Meinhart

**Affiliations:** Department of Biomolecular Mechanisms, Max Planck Institute for Medical Research, Heidelberg, Germany; Centre National de la Recherche Scientifique, France

## Abstract

During an interbacterial battle, the type-6-secretion-system (T6SS) of the human pathogen *Pseudomonas aeruginosa* injects the peptidoglycan(PG)-hydrolase Tse1 into the periplasm of Gram-negative enemy cells and induces their lysis. However, for its own benefit, *P. aeruginosa* produces and transports the immunity-protein Tsi1 into its own periplasm where in prevents accidental exo- and endogenous intoxication. Here we present the high-resolution X-ray crystal structure of the lytic enzyme Tse1 and describe the mechanism by which Tse1 cleaves the γ-D-glutamyl-l-*meso*-diaminopimelic acid amide bond of crosslinked PG. Tse1 belongs to the superfamily of N1pC/P60 peptidases but is unique among described members of this family of which the structure was described, since it is a single domain protein without any putative localization domain. Most importantly, we present the crystal structure of Tse1 bound to its immunity-protein Tsi1 as well and describe the mechanism of enzyme inhibition. Tsi1 occludes the active site of Tse1 and abolishes its enzyme activity by forming a hydrogen bond to a catalytically important histidine residue in Tse1. Based on our structural findings in combination with a bioinfomatic approach we also identified a related system in *Burkholderia phytofirmans*. Not only do our findings point to a common catalytic mechanism of the Tse1 PG-hydrolases, but we can also show that it is distinct from other members of this superfamily. Furthermore, we provide strong evidence that the mechanism of enzyme inhibition between Tsi1 orthologues is conserved. This work is the first structural description of an entire effector/immunity pair injected by the T6SS system. Moreover, it is also the first example of a member of the N1pC/P60 superfamily which becomes inhibited upon binding to its cognate immunity protein.

## Introduction

Systems evoking cell death are widespread in bacteria [1],[2], serving a multitude of different functions such as in virulence and biofilm formation [3], as weapons in the competition for biological niches [4],[5],[6] and to ensure stable maintenance of genetic elements [7] or as phage defense systems [8]. Most of these systems are classical two-component systems in which one component is an enzyme that interferes with cell vitality and can eventually kill bacteria (either called the effector protein or toxin) and the other a counteracting protein that can inhibit the toxic activity (immunity protein or antitoxin) [9]. Depending on whether the toxin is released or stays in the cell, these systems can be grouped into two classes: bacteriocins and classical toxin-antitoxin (TA) systems. Bacteriocins are effector proteins that are released from the bacterium in order to kill competing cells. Classical toxins remain in the host cell and so can cause cellular suicide [6],[7].

These classical TA-systems consist of a proteolytically stable toxin and an antitoxin that is less stable. Once *de novo* synthesis of the TA-system is impaired, the toxin is freed from its antitoxin by continuous proteolytic degradation and thereby induces cell death or stasis [7],[9],[10],[11]. Strikingly, the mechanisms by which toxin proteins promote cell death are quite diverse; among the toxin proteins are ribonucleases [12],[13], DNA gyrase inhibitors [7],[14], kinases that interfere with the translation machinery [15] or enzymes that impair peptidoglycan (PG) synthesis [16]. In contrast to the antitoxins of classical TA-systems which only neutralize their toxins temporarily, *i.e.*, until they are proteolytically degraded, their equivalent, the immunity proteins of bacteriocins, are thought to counteract toxic effector activity permanently. Moreover, the effector proteins are released by the bacteria in order to kill neighboring individuals which have not acquired immunity [4],[6]. As with TA-systems, the mechanisms by which these systems kill competing cells are diverse. For instance, the well-studied Barnase – Barstar system from *Bacillus amyloliquefaciens* includes a ribonuclease [17]; or the well studied Colicin toxin from *Escherichia coli* which is translocated through the cellular membrane into the cytosol of competing cells and exerts its toxic DNase- and RNase activity if no immunity protein is present to ensure resistance [18],[19],[20].

Recently, the type-6-secretion system (T6SS) was shown to be an injection machinery for effector proteins in Gram-negative bacteria that originally was described as a system that supports virulence of pathogenic bacteria [21],[22]. However, accumulating evidence suggests that it plays also a fundamental role in bacterial competition by injecting effector proteins into neighboring cells [23],[24]. A minimal T6SS apparatus is formed by 13 conserved core components but depending on the individual species up to 20 proteins assemble in the entire injection machinery [25],[26]. Especially the conserved core components share structural similarity with proteins of bacteriophage injection systems, indicating that T6SS is derived from phages [25],[27],[28]. Most importantly, the T6SS from the Gram-negative, pathogenic bacterium *Pseudomonas aeruginosa* was shown to inject at least three different effector molecules (named Tse1, Tse2, and Tse3; Type-6-Secretion-Exported) into the periplasmic space of competing cells [24]. Tse1 was shown to be an amidase that cleaves the γ-D-glutamyl-l-*meso*-diaminopimelic acid amide bond of crosslinked PG, and Tse3 is a muramidase that cleaves the PG-backbone between the *N*-acetylmuramic acid and the *N*-acetylglucosamine moieties [24]. Whilst the effector proteins Tse1 and Tse3 are dispensable for cellular growth, Tse2 is an essential protein in *P. aeruginosa* which functions by a yet unknown toxic mechanism [29]. Notably, Tse1 and Tse3 remain in the cytosol of the host cell and are separated from the PG by the cell membrane [24]. As injection of effector proteins by the T6SS would also cause cell death of siblings, *P. aeruginosa* co-synthesizes the cognate immunity proteins Tsi1 and Tsi3 (Type-6-Secretion-Immunity) and shuttles them into their periplasmic space to confer resistance [24]. In contrast, the third effector protein, Tse2 is toxic in the cytosol and relies on the presence of the cytosolic immunity protein Tsi2 which avoids self-intoxication [29].

Since interference with effector/immunity protein interaction would allow targeted killing of specific bacterial cells, these systems are of enormous interest for new antimicrobial approaches. However, the Tse/Tsi effector immunity systems from *P. aeruginosa* remain poorly understood on a structural level, with only three-dimensional models of the Tsi2 immunity protein having been reported to date [29],[30]. For this reason, we set out to determine the three-dimensional structure of the Tse1 effector protein to be able to explore its mechanism of toxicity on a structural level. Also, we have obtained the first atomic model of Tse1 in complex with its immunity protein Tsi1. Finally, we provide evidence that a similar Tsi1/Tse1 system also exists in other bacteria and that the mechanisms of their toxicity and its inhibition appear to be conserved.

## Results and Discussion

### The Tse1-effector is a *bona fide* N1pC/P60 cystein peptidase

We crystallized *P. aeruginosa* Tse1 and obtained excellent data, for which experimental structure factor phases were obtained from a Single-wavelength Anomalous Diffraction (SAD) experiment on selenomethionine-substituted protein crystals. Subsequently, the model was refined against 2.6 Å resolution data (Table 1). Interestingly, native Tse1 crystallized under similar conditions but with a different morphology and with a different crystal packing. Thus, phases for the native crystal form were obtained by molecular replacement using the refined model from the selenomethionine-substituted protein structure and refined to 1.7 Å resolution (Table 1). In both structures, four molecules per asymmetric unit were found which were virtually identical to each other (Table S1). Both models show excellent stereochemistry, with 96% of the residues in the preferred regions of the Ramachandran plot. Statistics of data collection, structure refinement and model quality are reported in Table 1.

**Table 1:** Data collection and refinement statistics.^a^

Crystal	Tse1 SeMet	Tse1 native	Tse1/Tsi1 phasing	Tse1/Tsi1 refinement
**Wavelength (Å)**	0.9794	0.9796	0.9794	0.9794
**Spacegroup**	*P*2_1_	*P*2_1_	*P*4_1_22	*P*4_1_22
**Cell parameters**	*a = *39.21, *b* = 108.40, *c* = 83.76, *β* = 93.80	*a = *48.24, *b* = 100.38, *c* = 62.18, *β* = 99.35	*a = 97.58*, *b* = 97.58, *c* = 423.53	*a = 97.56*, *b* = 97.56, *c* = 423.74
**Resolution (highest shell) (Å)**	50.0-2.6 (2.7-2.6)	50.0-1.7 (1.8-1.7)	50.0-3.4 (3.6-3.4)	50.0-3.2 (3.4-3.2)
**# reflections, all**	199,532 (19,251)	228,157 (36,107)	1,492,929 (240,447)	504,102 (83,437)
**# reflections, unique**	21,440 (2,189)	63,172 (9,887)	29,386 (4,519)	35,044 (5,664)
**Completeness (%)**	99.5 (95.9)	98.4 (97.8)	99.9 (100.00)	99.9 (99.9)
**Redundancy**	9.3 (8.8)	3.6 (3.7)	50.0 (53.0)	14.4 (14.7)
**I/σ(I)**	22.0 (8.4)	13.5 (3.1)	27.7 (13.6)	17.0 (6.5)
**R_merge_**	0.092 (0.333)	0.054 (0.469)	0.166 (0.454)	0.138 (0.450)
**Resolution range in refinement**	45.5-2.6	47.6-1.7		
**R_work_/R_free_**	0.206/0.251	0.174/0.202		0.224/0.263
**# atoms**				
Protein	4,392 (4 chains)	4,517 (4 chains)		8,896 (8 chains)
Water	127	330		
Ions	1 (1 Cl^−^), 9 (3 SCN^−^)	3 (3 Cl^−^), 6 (2 SCN^−^)		
Others		12 (2 glycerol)		
**Average B-factors**				
Protein	26.6	30.3		65.5
Water	26.9	35.1		
Ions	35.4	31.4		
Others		57.9 (glycerol)		
**Rmsd from target values**				
Bond lengths (Å)	0.008	0.008		0.006
Bond angles (°)	1.103	0.995		0.970
**%age of residues in region of Ramachandran plot :**				
Preferred	96.4	96.3		93.2
Allowed	3.6	3.0		6.7
Outliers	0.0	0.7		0.1

a Statistics calculated considering Friedel mates as identical reflections.

**Figure 1:**
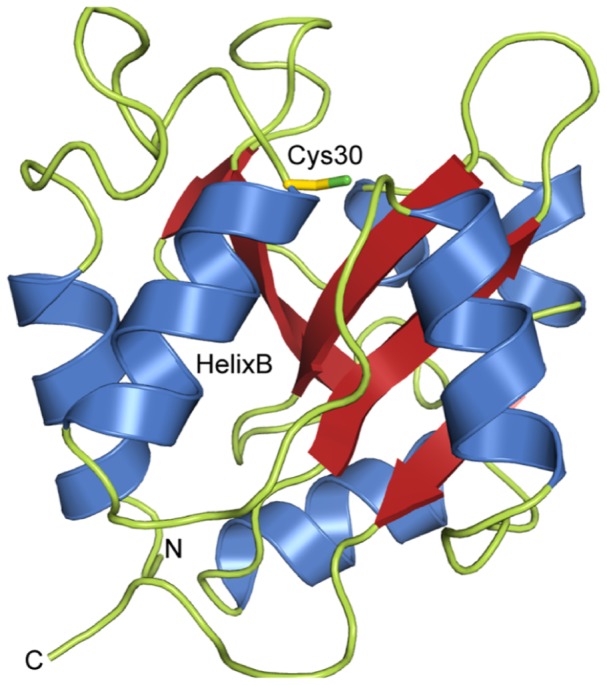
Crystal structure of *Pseudomonas. aeruginosa* Tse1. Crystal structure of *P.aeruginosa* Tse1. The central antiparallel β sheet (red) is surrounded by six α-helices (blue). Cys30, located at the beginning of helixB, is shown as a stick model.

Tse1 adopts the fold of N1pC/P60 papain-like cysteine peptidases, which are widespread among bacteria and of which homologues were identified in eukaryotes, phages, viruses, and archaea [31],[32],[33]. The core of the protein is formed by a twisted, five-stranded antiparallel β-sheet in a half-barrel configuration. This half-barrel is shielded by surrounding α-helices (Figure 1). A Dali search [34] for structurally similar proteins identified, amongst others, the N1pC/P60 domains of the γ-D-glutamyl-L-diamino acid endopeptidases YkfC from *Bacillus cereus* [35] (r.m.s.d. of 3.0 Å and 13% sequence identity), AvPCP from *Anabaena variabilis* [36] (r.m.s.d. of 3.2 Å and 15% sequence identity), NpPCP from *Nostoc punctiforme* [36] (r.m.s.d. of 3.1 Å and 12% sequence identity) and the Spr membrane-anchored cell wall hydrolase from *E. coli* [37] (r.m.s.d. of 3.0 Å and 16% sequence identity). Although the amino acid sequence identity to these hydrolases is very low, their central cores are structurally highly similar. The main structural differences are found close to the active site, which most likely reflects different modes of substrate binding and substrate specificity. Amongst these related enzymes, Tse1 seems to be unique as it is the only single domain enzyme whereas the others contain additional domains within their polypeptide chain. These additional domains are thought to target the proteins in different cellular compartments [36]. Most likely, Tse1 does not require such targeting domains as it remains in the cytosol of *P. aeruginosa* and seems to be exclusively exported by the T6SS [24].

**Figure 2:**
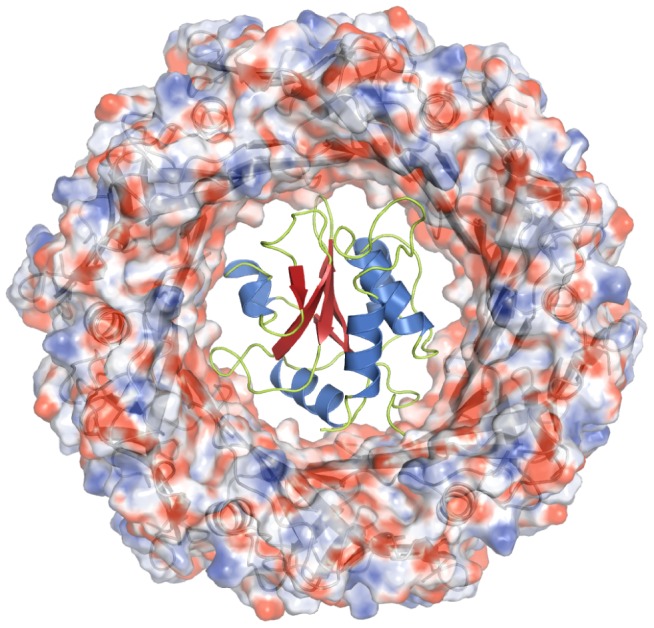
Model of Tse1 fitting through the Hcp1 ring of the T6SS injection needle. The hexameric Hcp1 ring (PDB: 1Y12) is shown as surface representation colored according to the electrostatic surface potential (contouring from +15 kT/e in blue to −15 kT/e in red). The ribbon representation of Hcp1 polypeptide chains is illustrated in gray underneath. The best-fit model of Tse1 is illustrated as ribbon representation using the color scheme from Figure 1.

Therefore, we wondered whether Tse1 can be exported through the injection needle of the T6SS in its functional conformation or whether only an unfolded/partially folded polypeptide chain can pass through the pore. The main component of this needle through which all effector proteins are secreted is formed by the Hcp (haemolysin co-regulated) protein [25],[27]. Structural studies on the Hcp1 protein from *P. aeruginosa* revealed that these injection needle forming polypeptide chains oligomerize into hexameric rings that have an internal diameter of 40 Å [28]. In fact, the maximal diameter of Tse1 is around 39 Å, suggesting that Tse1 can pass through the injection needle. Moreover, we could model Tse1 inside the pore of Hcp1 (Figure 2) supporting that T6SS can inject the Tse1 effector protein in its fully functional state.

### The active site architecture of Tse1

**Figure 3:**
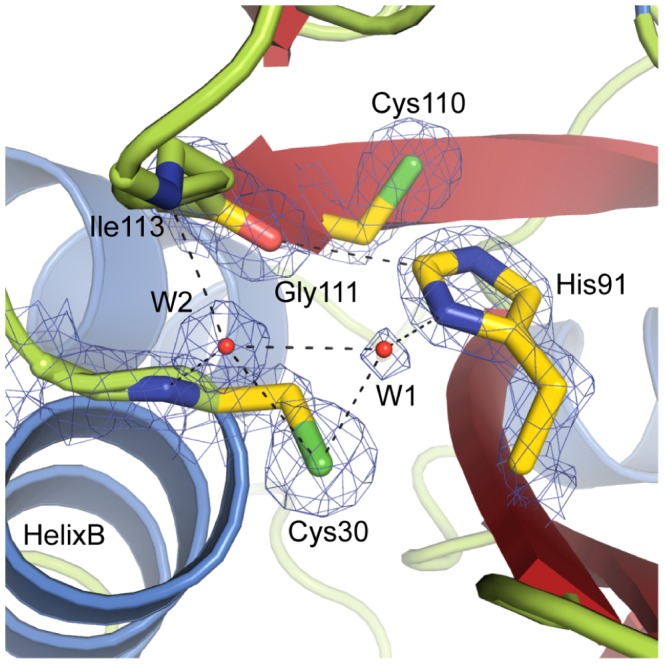
The active site of *Pseudomonas. aeruginosa* Tse1. Close-up of the active site of Tse1, indicating the catalytic diad Cys30-His91, as well as Ile113, Gly111 which are proposed to be catalytically important, and the water molecules W1, which is surmised to play a role in cysteine deprotonation, as well as W2, located in the oxyanion hole. Hydrogen bonds are shown as black dashed lines, and the refined 2*m*F_o_-DF_c_ electron density map is shown at a contour level of 2.0 σ. Also shown is Cys110, in the position where a third catalytic residue was proposed for N1pC/P60 papain-like cysteine peptidases to which Tse1 is structural related.

In analogy to the related papain/cathepsin proteases, Tse1 contains a catalytically important cysteine residue that is required for Tse1 toxicity [24]. This Cys30 is part of a highly conserved amino acid stretch [24],[31] that is located in a shallow cleft at the surface of Tse1 at the beginning of helix B (Figure 3). Cys30 is found to be in close proximity to a histidine residue (His91, see Figure 3), which is the second highly conserved residue within the N1pC/P60 family [31]. Together, Cys30 and His91 form the catalytic diad commonly found in cysteine proteases [38]. For the cysteine proton to be abstracted by the Nδ of the histidine residue, His91 has to be in its normal tautomeric state (Nε2-H) [39]. Most likely, the deprotonation of the cysteine residue is performed via a water molecule (W1) which is bound between Cys30 and His91 rather than by the histidine residue directly (Figure 3). The resulting thiolate anion is stabilized by the dipole of helix B [40]. Furthermore, the Cε1 of the histidine residue is within hydrogen bonding distance to the carbonyl oxygen of Gly111 which serves to stabilize the imidazolium form of His91 (Figure 3), as already reported for serine proteases [41]. The highly reactive thiolate of Cys30 performs a nucleophilic attack on the γ-D-glutamyl-l-*meso*-diaminopimelic acid amide bond of crosslinked PG, resulting in a tetrahedral oxyanion intermediate bound by the so called oxyanion hole [42] formed by the backbone amides of Cys30 and Ile113. In our crystal structure of Tse1, a water molecule (W2) was found at this position (Figure 3). The tetrahedral intermediate collapses and an acylenzyme is formed. Subsequently, the leaving group is protonated by the imidazolium of His91 and the amine is released. A catalytic water molecule then perfoms a nucleophilic attack splitting the acylenzyme after which the product is released and the enzyme is ready for another round of catalysis.

Recently, a second histidine residue has been proposed to be involved in the mechanism of the closely related *E. coli* Spr hydrolase [37]. In fact, the Nδ1 of this second histidine residue (His131) was found to form a hydrogen bond to the Nε2 proton of the imidazole moiety of the Cys-His diad and both histidine residues adopt the Nε2H tautomeric state (Figure S1). Based on this observation and supported by bioinformatic studies, the N1pC/P60 family had been suggested to make use of a conserved catalytic triad instead of a diad, as in serine proteases, where the third conserved residue was proposed to maintain the Nδ1H tautomeric state of the histidine through interactions with its carboxylate [43]. This hypothesis was further supported by structural reports, which described the existence of this third histidine residue as well [35],[36]. However, in Tse1 we observed a cysteine residue at this position, which cannot maintain the correct tautomeric form of His91 with a hydrogen bond (Figure 3), demonstrating that the minimal requirement for N1pC/P60 hydrolases is a Cys-His catalytic diad. Thus, the catalytic mechanism is far more similar to that of classical papain/cathepsin enzymes than was previously anticipated.

### A homologue of the Tse1/Tsi1 system in *Burkholderia phytofirmans*

In order to verify whether the enzymatic activity of Tse1 relies on a catalytic diad or triad we performed an amino acid similarity search [44] using the NCBI non-redundant database focusing on the conservation of catalytically important residues. However, we almost exclusively identified Tse1 proteins in various *P. aeruginosa* strains with at least 93% to 99% amino acid sequence identity (see Table S2). Moreover, not only are the amino acid sequences highly similar but this high degree of conservation was also present at genomic level with only very few point mutations between the different strains; suggesting relatively recent horizontal spreading of the allele through the strains of *P. aeruginosa*. Predicted proteins that showed less conservation were found as well, but most of them were assigned as N1pC/P60 proteins. In order to identify further *bona fide* Tse1/Tsi1 systems in other organisms, we searched the genomic regions upstream and downstream of the Tse-like ORFs for the putative Tsi1 orthologues in order to identify ORFs encoding for a Tsi1 related immunity protein within a bicistronic operon (see Table S2). Indeed, we identified a number of highly conserved Tse1/Tsi1 encoding operons with similar architectures in various *P. aeruginosa* strains. As with Tse1, these Tsi1 ORFs were nearly identical at the genomic level. Moreover, all these operons showed a bicistronic arrangement in which Tsi1 was encoded upstream of Tse1 and both ORFs were separated by a spacer sequence.

**Figure 4:**
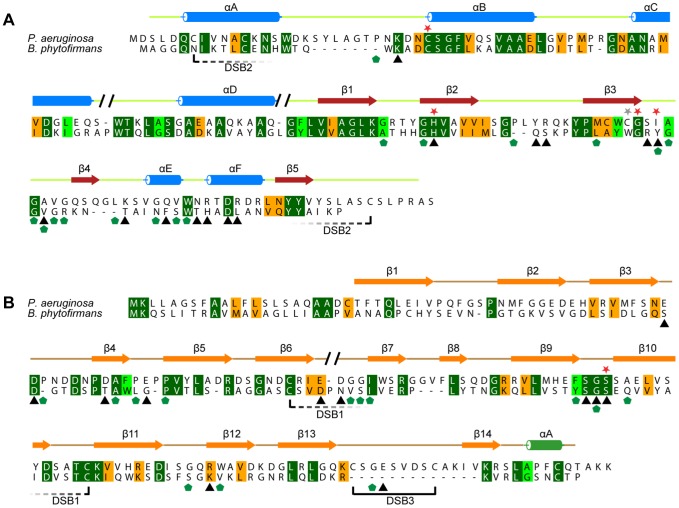
Amino acid sequence alignment of Tse1 and Tsi1 of P*seudomonas aeruginosa* and *Burkolderia phytofirmans*. Amino acid sequence alignment of *P. aeruginosa* and *B. phytofirmans* Tse1 (A) and Tsi1 (B). Helices and β-strands in Tse1 are colored blue and red, respectively. Helices in Tsi1 are colored in green and β-strands are shown in orange. Red stars show either residues of Tse's active site or residues in Tsi1 which are important for catalytic inhibition of Tse1. A grey stars indicates Cys110 located at the structurally equivalent position of the histidine residue in the catalytic triad of N1pC/P60 papain-like cysteine peptidases. Below the sequence alignments, residues which connect Tse1 and Tsi1 in our crystal structure by either salt bridges and hydrogen bonds (black triangles) or hydrophobic interactions (green pentagons) are indicated. Dashed lines and solid lines represent disulfide bond (DSB) formation. Conservation of amino acids goes from dark green (high conservation) to orange (low conservation).

However, we found a less conserved orthologue of Tse1 in the genome of *Burkholderia phytofirmans* (NCBI accession code YP_001888916.1) which had 34% amino acid sequence identity with Tse1 from *P aeruginosa* (Figure 4A). This Tse1-related protein seems to be a *bona fide* Tse1 effector protein, since it is encoded together with its immunity protein Tsi1 (NCBI accession code YP_001888915.1; 30% amino acid sequence identity with *P. aeruginosa* Tsi1 (see Figure 4B) from a bicistronic operon. When comparing the operon architecture of the Tse1/Tsi1 system from *P. aeruginosa* with that from *B. phytofirmans*, we found that the ORF arrangement had been swapped. In contrast to the genome of *P. aeruginosa*, in *B. phytofirmans* Tse1 is encoded upstream from Tsi1. Furthermore, the translational start codon AUG of the Tsi1-ORF overlaps with the opal stop codon of the Tse1-ORF and the two ORFs are not separated by any spacer sequence, suggesting that DNA shuffling events have lead to a different operon arrangement. This hypothesis is further supported by an independent and very recent bioinformatical survey which identified various Tse1-analogous but not homologous variants in a plethora of β- and γ-proteobacteria [45]. Whereas, the effector proteins seemed to be conserved, their cognate immunity proteins differ significantly, suggestive of a highly dynamic nature of the effector-immunity operon.

Our finding enabled us to rule out that a catalytic triad is the common active site of all N1pC/P60 protein as previously suggested [35],[36], [37] since in the amino acid sequence of *B. phytofirmans* Tse1 a tryptophan residue is located at the structurally equivalent position to the cysteine residue in *P. aeruginosa* Tsi1 (Figure 4A) which also cannot be involved in stabilization of the tautomeric state of His91 by establishing a hydrogen bonding network.

### Inhibition of Tse1 by Tsi1

To investigate how the Tse1 effector protein from *P. aeruginosa* is inhibited by its immunity protein Tsi1 we co-crystallized Tse1 and Tsi1 lacking the predicted leader sequence for periplasmic localization and determined the structure of the Tse1/Tsi1 complex at 3.2 Å resolution by a SAD experiment on selenomethionine-substituted protein crystals. Statistics of data collection, structure refinement and model quality are reported in Table 1.

**Figure 5:**
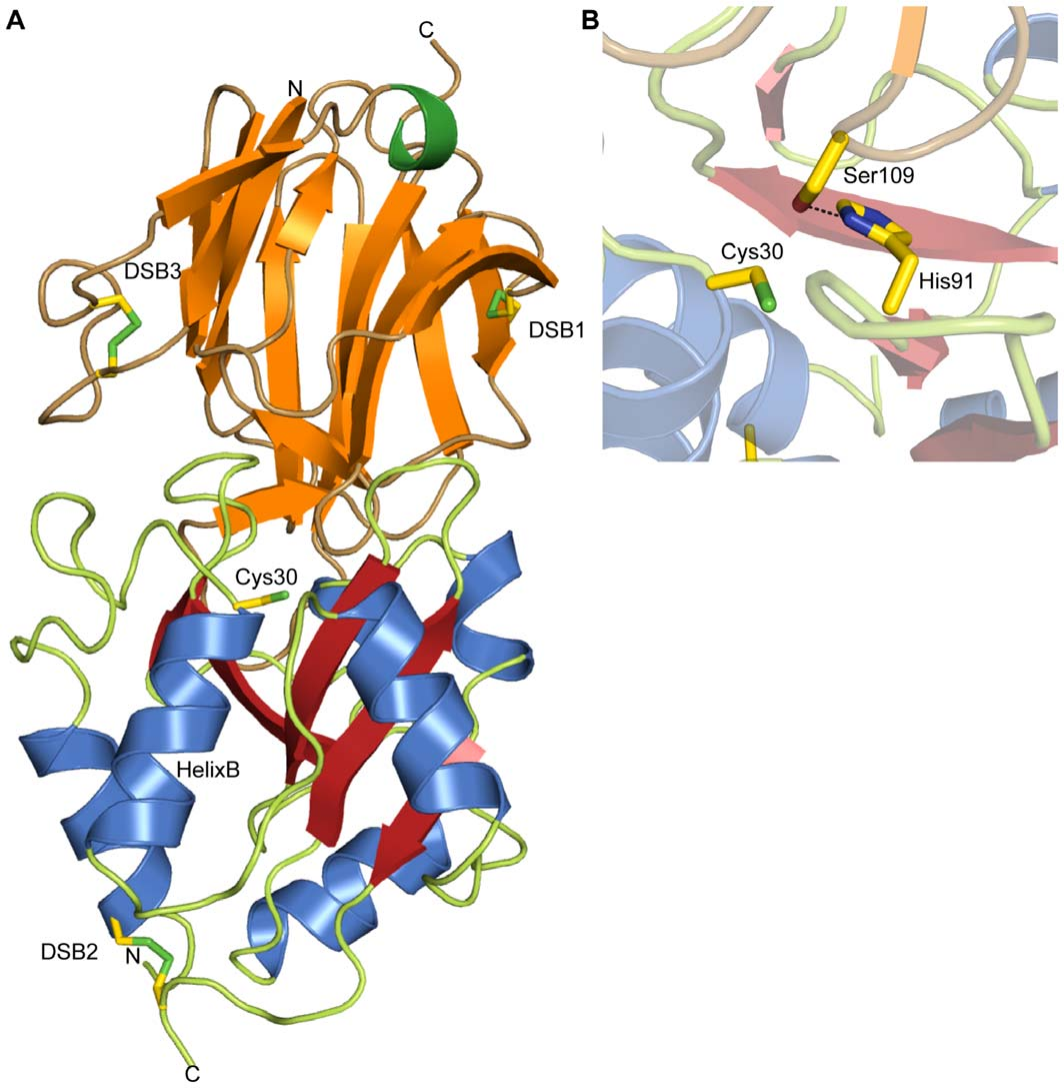
Crystal structure of the Tse1/Tsi1 complex and inhibition of Tse1 by Tsi1. (A) Crystal structure of the P*seudomonas aeruginosa* Tse1/Tsi1 complex using the color scheme of figure 1 for Tse1. Tsi1 is formed by three antiparallel β-sheets (orange) arranged as a partial β-propeller, and a short C-terminal α-helix (green). The N-terminal β-sheet consists of the β-strands β1, β2, β3, β5, and β6. The central β-sheet is made of the β-strands β1, β8, β9, β10, and β11. The C-terminal β-sheet is formed by the β-strands β12, β13, and β14. Residues and disulfide bonds (DSB) in Tse1 (DSB2) as well as in Tsi1 (DSB1 and 3) are depicted as sticks. (B) Close-up of the interaction between Tse1 and Tsi1 at the Tse1 active site. Tsi1 inserts the Ser109 side chain into the Tse1 active site, forming a hydrogen bond to the catalytic His91, keeping it from deprotonating Cys30.

Tsi1 is formed by three twisted antiparallel β-sheets resembling a fragmented β-propeller [46] followed by a short C-terminal α-helix (Figure 5A). The first two β-sheets are highly interlinked with each other. In particular, the N-terminal strand 1 crosses over these first two sheets in a β-addition module and a disulfide bond between Cys79 and Cys121 (DSB 1) forms a rigid covalent link between them (Figure 5A). In contrast, the third β-sheet loosely packs onto the rigid scaffold provided by the two N-terminal, tightly interacting sheets. Importantly, both cysteine residues of DSB 1 are conserved in the Tsi1 amino acid sequence from *P. aeruginosa* and *B. phytofirmans* (Figure 4B). A Dali search [34] for proteins that are structurally similar to Tsi1, almost exclusively identified the β-propeller unit of type IV dipeptidyl peptidases (DPP IV). The closest structurally related proteins are β-propeller units of DPP IV from the human pathogen *Stenotrophomonas maltophilia* [47] (r.m.s.d. of 2.6 Å and 10% sequence identity) and from *Homo sapiens* [48] (r.m.s.d. of 2.6 Å and 10% sequence identity). Since Tsi1 is a partial β-propeller we initially wondered whether we had artificially truncated Tsi1 by an incorrect assignment of the translational start codon. However, this is rather unlikely, since the Tsi1 translational start codon of *B. phytofirmans* is uniquely defined by the bicistronic operon architecture and the downstream region aligns very well with the assigned ORF in *Pseudomonas* (Figure 4B).

**Figure 6:**
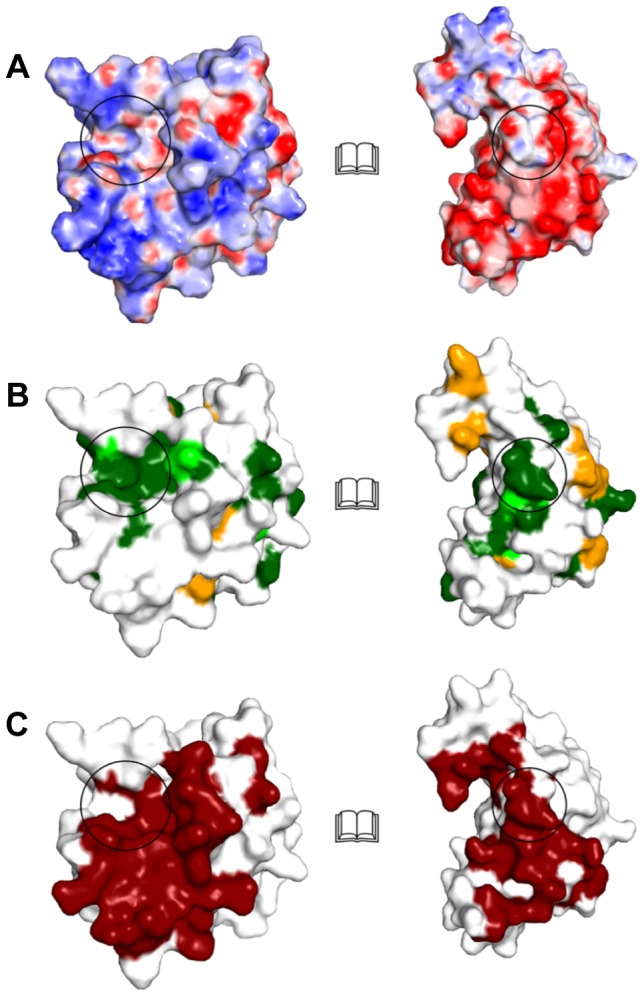
Surface representation of *Pseudomonas aeruginosa* Tse1 and Tsi1 showing their electrostatic potential, interaction and conservation. “Open-Book view” of the Tse1/Tsi1 complex showing surface representations of Tse1 (left) and Tsi1 (right). (A). Electrostatic surface potential of Tse1 and Tsi1 contoured over a range of ±15 kT/e (blue/red). (B) Amino acid sequence conservation (Figure 3) mapped onto the molecular surfaces in identical view as in (A). (C) Tse1/Tsi1interacting surface patch colored in red. The highly conserved interaction patches of Tsi1 and Tse1 are highlighted with black circles in (A), (B), and (C).

Tsi1 and Tse1 form a complex *via* a contact surface (on average 1049 Å^2^ for all four molecules per asymmetric unit) which occludes the active site of Tse1. When comparing the structures of Tse1 in complex with Tsi1 and Tse1 alone, no major conformational changes in the overall structure or even in the active site were observed. More importantly, the loop region between β-strands 9 and 10 of Tsi1 directly interacts with residues of the active site of Tse1. Moreover, the residues within this loop region are strictly conserved between *Pseudomonas* and *B. phytofirmans*, suggesting a common binding mode. Apart from steric hindrance of substrate binding by Tsi1, the hydroxyl function of Tsi1 Ser109 forms a hydrogen bond with the Nδ of the catalytic Tse1 His91. In doing so, it replaces the catalytically important water molecule (W1) and prevents deprotonation of Cys30 (Figure 5B). It is plausible that this hydrogen bonding network serves a dual function. First, it inhibits enzyme catalysis. Second, it prevents activation of Cys30 to a reactive thiolate anion, which then could readily be oxidized resulting in an irreversible inactivation of the enzyme. All other residues within this conserved loop regions form hydrogen bonds to Tse1 and thereby tether Tsi1 to the active site of Tse1. When comparing the molecular surface of Tse1 and Tsi1 and its conservation, we found a perfect complementary of the two molecular surfaces and this conserved loop region between β-strands 9 and 10 forms a knob on the molecular surface of Tsi1 which is inserted into the active site (Figure 6). Furthermore, a closer inspection of the molecular interactions of Tsi1 and Tse1 and in particular of the electrostatic potential mapped onto the molecular surfaces reveals that the contact surfaces are complementary not only in shape, but also in electric charge (Figure 6A). Moreover, as shown in Figure 6B, a surface patch involved in Tse1/Tsi1 interactions consist of conserved residues.

### Stabilization of Tse1 and Tsi1 by disulfide bridges

When comparing the structures of native Tse1 and selenomethionine-substituted protein which displayed different crystal packings, we found that the selenomethionine-substituted protein of Tse1 had formed a disulfide bond between Cys7 and Cys148. This disulfide bond (DSB 2, see Figure 4A and Figure 5A) covalently connects the N-terminal helix A and the very C-terminal region. However, in the native protein DSB2 was absent. Since DSB2 was observed in the highly redundant SAD data, collected at the selenium peak, its absence in the native structure is unlikely to be caused by radiation damage. Moreover, both variants were expressed using a cytosolic expression construct and were thus not exported into the periplasm; a cellular regime which normally would support disulfide-bond formation in *E. coli*. Also, during the entire purification and crystallization procedure reducing agents were present in both cases. However, once we excluded any reducing agents from the purification procedure of native Tse1 protein and setup crystallization experiments, crystals with similar morphology as observed for selenomethionine-substituted proteins were observed suggesting that in those crystals DSB2 had been formed too. Unfortunately, the diffraction quality of those crystals was very poor and we could not collect data that would allow structure refinement. Finally, this disulfide bond was observed in the crystal structure of Tse1 in complex with Tsi1 as well (Figure 5A).

Thus, we argue that DSB2 formation or not is not an experimental artifact. Indeed, it is possible that in cytosolic Tse1 DSB2 is open, and that it closes after transportation into the periplasmic space by T6SS. It remains to be verified what the functional relevance of formation of this covalent linkage is. Most likely, it serves structural and proteolytic stability in the periplasm. In fact, it is rather unlikely that it could have any functional importance on the enzymatic activity, since Tse1 from *B. phytofirmans* lack both cysteine residues (Figure 4A).

Such structural disulfide bonds were observed in Tsi1 as well. DSB 1 tethers the N-terminal to the middle β-sheet as discussed above. Furthermore, a second disulfide bond (DSB 3) was observed in the loop region connecting strand 13 and 14 where Cys147 and Cys155 form a covalent short-cut (Figure 5A). Most likely, as in Tse1 this posttranslational modification serves to stabilize Tsi1 in the periplasmic space. However, as with Tse1, this disulfide bond is not present in the Tsi1 orthologue of *B. phytofirmans* (Figure 4B).

### Conclusion

The current work showed that Tse1 is a cysteine peptidase structurally related to the N1pC/P60 hydrolase superfamily, which are enzymes involved in peptidoglycan degradation and recycling. Tse1 is unique as it is a single domain protein, lacking the additional domains which in other N1pC/P60 hydrolases serve as cellular localization modules. Moreover, Tse1 is secreted into the periplasm by the T6SS and most likely does not require localization domains. Once Tse1 arrives in the periplasm of *P. aeruginosa*, whether injected by a neighboring T6SS containing cell or having strayed from the cell's own cytosol, the immunity protein Tsi1 is prepared to bind and inhibit Tse1 to neutralize its detrimental activity. By binding to a large surface patch of Tse1 it occludes the active site and specifically inhibits enzyme activity forming a hydrogen bond to His91 of the catalytic diad. Furthermore, we could show that Tse1 makes use of a Cys/His catalytical diad whereas a third residue, which was previously proposed to be relevant for other members of these PG hydrolases is missing. Finally, we have evidence that the Tse1/Tsi1 defense mechanism is not only restricted to *P. aeruginosa* but can also be found in the closely related bacterium *B. phytofirmans*.

## Materials and Methods

### Construct design

DNA of ORFs encoding full-length Tse1 lacking the stop codon and Tsi which lacks the leader sequence for periplasmic localization was amplified by PCR from *P. aeruginosa PAO1* chromosomal DNA using the following primers: tse1_ndeI_f; tse1_notI_r; tsi1_dN23_ncoI_f and tsi1_mfeI_r (see Table S3). In order to generate a bicistronic co-expression construct, Tse1 and Tsi1 were ligated into a modified pET21d plasmid [49] harboring two ribosomal binding sites. Tsi1 was inserted between the NcoI/EcoRI sites, whereas Tse1 was integrated between the NdeI/NotI sites, resulting in a C-terminal hexahistidine fusion tag. The construct was verified by DNA sequencing.

### Protein expression and purification

For expression of native proteins, *E. coli* BL21-CodonPlus(DE3)-RIL cells (Stratagene) were transformed with the bicistronic expression construct (see above) and grown at 310 K in LB-medium containing ampicillin (100 µg/ml) and chloramphenicol (34 µg/ml) until mid-log phase (OD_600_∼0.6). Subsequently, the temperature was reduced to 293 K and protein expression was induced by addition of 0.5 mM isopropyl β-*D*-1-thiogalactopyranoside. Cells were harvested 16 hours after induction by centrifugation and resuspended in buffer A (50 mM Tris-HCl pH 8.0, 300 mM NaCl, 5 mM 2-mercaptoethanol). Cells were lysed by sonication and cell debris was removed by centrifugation at 20,000×g. The clarified supernatant was loaded onto Ni-NTA agarose (Qiagen) equilibrated with buffer A. After washing the column resin with buffer A, the bound proteins were eluted with buffer B (50 mM Tris-HCl pH 8.0, 300 mM NaCl, 500 mM imidazole, 5 mM 2-mercaptoethanol). The proteins were dialyzed overnight against 400 volumes of buffer C (50 mM Tris-HCl pH 8.0, 200 mM NaCl, 1 mM ethylenediaminetetraacetic acid (EDTA), 2 mM dithioerythritol (DTE)) using a 5,000 MWCO dialysis tube (Roth). The dialyzed proteins were diluted 1∶10 with buffer D (50 mM Tris-HCl pH 8.0, 2 mM DTE) and loaded onto a MonoQ 10/100 GL column (GE Healthcare) equilibrated with buffer D. The flow-through which exclusively contained free Tse1 was collected and used in further purification steps (see below). The column was washed with buffer D supplemented with additional 20 mM NaCl. The bound Tse1/Tsi1 complex was eluted in a linear gradient of 10 column volumes to buffer E (50 mM Tris-HCl pH 8.0, 1 M NaCl, 2 mM DTE). Finally, the eluted Tse1/Tsi1 complex was concentrated and applied to a Superdex 75 10/300 GL column equilibrated with buffer F (50 mM HEPES-NaOH pH 7.5, 200 mM NaCl, 1 mM *tris*(2-carboxyethyl)phosphine). The protein complex was judged by Coomassie stained SDS-PAGE to be 99% pure and the eluted fractions were concentrated by ultrafiltration to 10 mg/ml as determined by the absorbance at 280 nm (ε_280_ = 46,980 cm^−1^ M^−1^).

To concentrate and further purify free Tse1, the flow-through from the anionic exchange chromatography step was reloaded onto a Ni-NTA agarose column using the same protocol as described for the initial Ni-NTA purification step. The eluted Tse1 protein was concentrated and applied to a Superdex 75 10/300 GL column (GE Healthcare) equilibrated with buffer F. The eluted fractions containing Tse1 protein were judged to be 99% pure based on Coomassie stained SDS-PAGE and subsequently pooled and concentrated by ultrafiltration to ∼17 mg/ml as determined by the absorbance at 280 nm (ε_280_ = 32,440 cm^−1^ M^−1^); flash frozen in liquid nitrogen and stored at 193 K.

Selenomethionine-substituted Tse1 protein and Tse1/Tsi1 complex were expressed according to [50] and protein purification was performed essentially as described for the native proteins except that buffer A contained additional 50 mM (NH_4_)_2_SO_4_, buffer C, D, and E contained 5 mM DTE, and buffer F contained 3 mM *tris*(2-carboxyethyl)phosphine. Concentration and storage conditions for selenomethionine substituted proteins were similar to those of native proteins.

### Crystallization and cryo-protection

Crystals of Tse1 grew within one day at 293 K in a hanging-drop vapor diffusion setup using a 1∶1 of protein to reservoir ratio and 700 µl of reservoir solution containing 200 mM KSCN, 20% *(w/v)* PEG 3350, and 1% *(v/v)* MPD. Notably, crystals in dimension of 50×70×200 µm^3^ grew initially in the drop which started to decompose 2 to 3 weeks after crystal growth. Simultaneously with the disappearance of those crystals, new crystals of needle-shaped morphology appeared. However, for data collection only the crystals that initially grew from the native protein were used. In contrast, exclusively the crystals of needle-shaped morphology were observed for the selenomethionine-substituted Tse1 protein crystals and the concentration of PEG 3350 had to be reduced to 15% *(w/v)* in order to grow crystals that diffracted to sufficiently high resolution. Crystals of native as well as selenomethionine-substituted Tse1 protein crystals were soaked in the reservoir solution supplemented with additional 25 mM HEPES-NaOH pH 7.5 and 20% *(v/v)* glycerol for cryo-protection and subsequently flash-cooled in liquid nitrogen.

Co-crystals of the Tse1/Tsi1 complex grew within 3 days at 293 K in a hanging-drop vapor diffusion setup using a 1∶1 protein to reservoir ratio and 700 µl reservoir solution containing 100 mM sodium cacodylate pH 6.5, 250 mM (NH_4_)_2_SO_4_, and 18% *(w/v)* PEG 8000. In order to improve the diffraction quality of those crystals, a dehydration protocol was applied. Therefore, the crystals were cooled to 277 K and stepwise transferred into the reservoir solution containing 5% *(v/v)*, 10% *(v/v)*, and finally 15% *(v/v)* PEG 400. This final solution afforded sufficient cryo-protection and the crystals were flash-cooled in liquid nitrogen.

### Data collection and structure determination

All diffraction data were collected at the X10SA beam line of the Swiss Light Source, Villigen, at 100 K and processed with XDS [51]. For all datasets, 5% of the reflections were omitted during refinement and used for calculation of an R_free_ value. A highly redundant SAD dataset was collected from a selenomethionine-substituted Tse1 protein crystal, from which the heavy-atom substructure was determined using SHELXD [52]. Phasing with SHELXE [52] resulted in interpretable electron density into which the model was built using COOT [53] and refined using REFMAC [54]. Diffraction data of native proteins were phased by molecular replacement methods using the refined model of the selenomethionine-substituted structure and phases were extended and refined in cycles of manual building and refinement with REFMAC [54] using TLS [55] against a 1.7 Å dataset resulting in a high-resolution structure of excellent geometry.

A second, highly redundant SAD dataset was collected from a selenomethione-substituted Tse1/Tsi1 protein crystal, which was phased using the identical protocol as described above. After phase improvement with SHELXE [52] an interpretable electron density was obtained into which the high-resolution model of Tse1 could be placed. Tsi1 was built manually using COOT [53]. The initial model was improved using the annealing protocol of CNS [56],[57] and strict non-crystallographic symmetry was applied during refinement to compensate for the resolution. Once the model was complete it was further refined using REFMAC [54] with moderate non-crystallographic symmetry restraints and TLS [55] applied. The quality of all reported models was evaluated using MolProbity [58] and figures were prepared using PYMOL [59] and surface-potentials have been calculated using the APBS plug-in application [60]. Sequence alignments were illustrated using the program ALSCRIPT [61] and the sequence conservation was determined by the AMAS-server [62]. The superposition of individual polypeptide chains was performed using LSQKAB [63]. Molecular surface contacts were analyzed using the PISA server [64]. Atomic coordinates and structure factor amplitudes have been deposited in the Protein Data Bank (PDB) under accession code 4FGD (Tse1 SeMet), 4FGE (Tse1 native), and 4FGI (Tse1/Tsi1).

## Supporting Information

Figure S1 **Comparison of the Tse1 with the of Srp (PDB:2K1G) active site.** (A) Close-up view of the active site of Tse1 showing the catalytic Cys-His diad (see also Figure 3). (B) Close up view of the active site of *Escherichia coli* Srp (PDB: 2K1G) with its Cys-His-His triad in similar orientation.
(PDF)

Table S1 **Deviation for main chain atoms of Tse1 native and Tse SeMet.**
(PDF)

Table S2 **Tse1/Tsi1 systems in different** ***Pseudomonas aeruginosa*** **strains.**
(PDF)

Table S3 **Primer for Tse1 and Tsi1ΔN23 construct design.**
(PDF)
