# Supplementary figures and images for: Structural Insights into the Effector – Immunity System Tse1/Tsi1 from Pseudomonas aeruginosa
Source: PLoS One. 2012 Jul 6;7(7):e40453. doi: 10.1371/journal.pone.0040453 (PMC3391265; doi:10.1371/journal.pone.0040453)

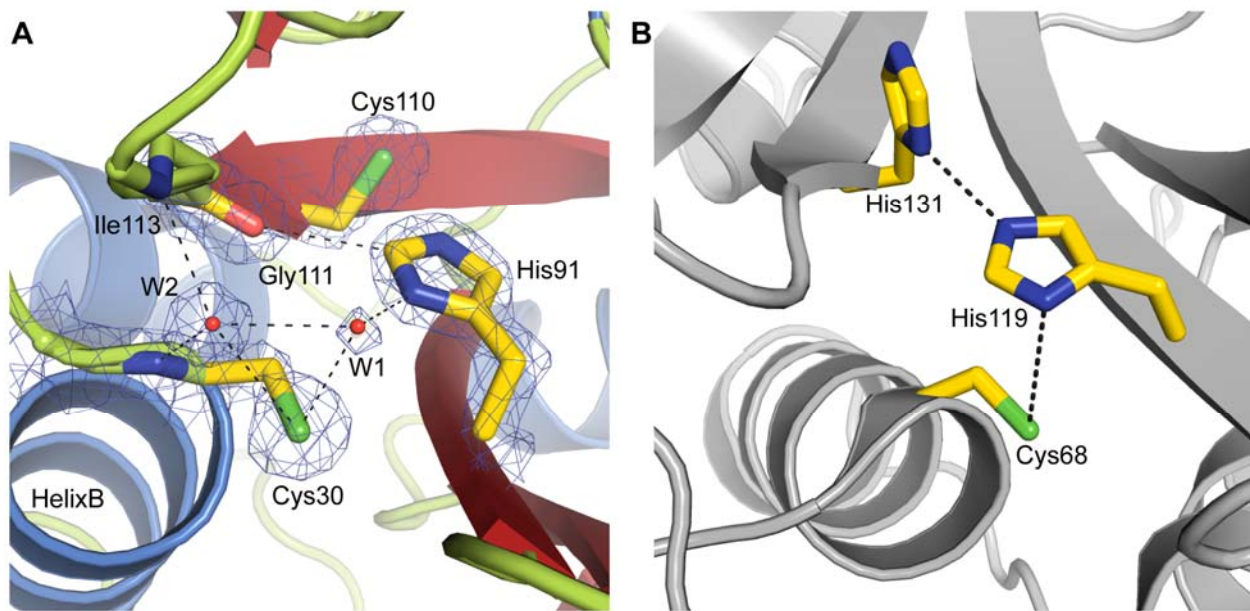

**FigureS1**

Supplement: Figure S1 — Comparison of the Tse1 with the of Srp (PDB:2K1G) active site. (A) Close-up view of the active site of Tse1 showing the catalytic Cys-His diad (see also Figure 3). (B) Close up view of the active site of Escherichia coli Srp (PDB: 2K1G) with its Cys-His-His triad in similar orientation. (PDF) [file pone.0040453.s001.pdf]
