# Supplementary material for: Structural Insights into the Effector – Immunity System Tse1/Tsi1 from Pseudomonas aeruginosa
Source: PLoS One. 2012 Jul 6;7(7):e40453. doi: 10.1371/journal.pone.0040453 (PMC3391265; doi:10.1371/journal.pone.0040453)
Supplement: Table S1 — Deviation for main chain atoms of Tse1 native and Tse SeMet. (PDF) [file pone.0040453.s002.pdf]

Table S1: Deviation for main chain atoms of Tse1 native[a] and Tse SeMet[b].

Tse1 native

	chainD on chainA	chainD on chainB	chainD on chainC
mean deviation (Å)	0.212	0.156	0.213
r.m.s.d. (Å)	0.326	0.214	0.333
max. deviation (Å)	2.985	2.617	2.553
min. deviation (Å)	0.009	0.011	0.014

Tse1 SeMet

	chainD on chainA	chainD on chainB	chainD on chainC
mean deviation (Å)	0.210	0.223	0.165
r.m.s.d. (Å)	0.243	0.256	0.221
max. deviation (Å)	1.470	1.010	1.947
min. deviation (Å)	0.027	0.033	0.016

[a] residues ranging from Ser3 to Leu150 were included in the superposition, except for chainD on chainB where residue Leu150 was excluded.

[b] residues ranging from Ser3 to Ser149 were included in the superposition.
