# Supplementary material for: Structural Insights into the Effector – Immunity System Tse1/Tsi1 from Pseudomonas aeruginosa
Source: PLoS One. 2012 Jul 6;7(7):e40453. doi: 10.1371/journal.pone.0040453 (PMC3391265; doi:10.1371/journal.pone.0040453)
Supplement: Table S2 — Tse1/Tsi1 systems in different Pseudomonas aeruginosa strains. (PDF) [file pone.0040453.s003.pdf]

Table S2: Tse1 / Tsi1 systems in different *Pseudomonas aeruginosa* strains.

P. aeruginosa	**Tse1 accession number**	**Tsi1 accession number**
PAO1	NP_250535.1	NP_250536.1
2192	ZP_04933560.1	ZP_04933561.1
PACS2	ZP_01365195.1	ZP_01365196.1
39016	ZP_07796430.1	ZP_07796431.1
sp. 2_1_26	ZP_09053571.1	ZP_09053570.1
UCBPP-PA14	YP_791397.1	YP_791396.1
PAb1	ZP_06879238.1	ZP_06879237.1
PADK2_CF510	EIE44393.1	EIE44322.1
138244	EGM22773.1	EGM22744.1
PA7	YP_001348808.1	YP_001348807.1
