# Supplementary material for: Structural Insights into the Effector – Immunity System Tse1/Tsi1 from Pseudomonas aeruginosa
Source: PLoS One. 2012 Jul 6;7(7):e40453. doi: 10.1371/journal.pone.0040453 (PMC3391265; doi:10.1371/journal.pone.0040453)
Supplement: Table S3 — Primer for Tse1 and Tsi1ΔN23 construct design. (PDF) [file pone.0040453.s004.pdf]

Table S3: Primer for Tse1 and Tsi1ΔN23 construct design.

Primer	Sequence
tse1_ndeI_f	5’-CGCAACATATGGACAGTCTCGATCAATGCATCGTC-3’
tse1_notI_r	5’-GCTGCGGCCGCACTGGCCCTGGGCAGGCTGCAAC-3’
tsi1_dN23_ncoI_f	5’-GCAACCATGGCTTTCACCCAGCTGGAAATCGTG-3’
tsi1_mfeI_r	5’-GCTCAATTGTCATTTCTTTGCGGTCTGGCAGA-3’
